# Hospitals implementing changes in law to protect children of ill parents: a cross-sectional study

**DOI:** 10.1186/s12913-018-3393-2

**Published:** 2018-08-06

**Authors:** Bjørg Eva Skogøy, Knut Sørgaard, Darryl Maybery, Torleif Ruud, Kristin Stavnes, Elin Kufås, Gro Christensen Peck, Eivind Thorsen, Jonas Christoffer Lindstrøm, Terje Ogden

**Affiliations:** 1 Nordland Hospital Trust, Kløveråsveien 1, 8092 Bodø, Norway; 2 The Faculty of Health Sciences, UiT, The Arctic University of Norway, Box 6050, 9037 Tromsø, Norway; 3 Monash University Department of Rural Health, Box 973, Moe, VIC 3825 Australia; 4 Department for Research and Development, Mental Health Services, Akershus University Hospital, Box 1000, 1478 Lørenskog, Norway; 5 Institute of Clinical Medicine, University of Oslo, Box 1171, Blindern, 0318 Oslo, Norway; 6 Vestre Viken Hospital Trust, Box 800, 3004 Drammen, Norway; 7 Stavanger University Hospital, Box 8100, 4068 Stavanger, Norway; 8 BarnsBeste (Children’s Best Interests) - National Competence Network for Children as Next of Kin, Sørlandet Hospital Trust, Box 416, 4604 Kristiansand, Norway; 9 Norwegian Center for Child Behavioral Development, Unirand, Box 7053, Majorstuen, 0368 Oslo, Norway; 10 Institute of Psychology, University of Oslo, Box 1171, Blindern, 0318 Oslo, Norway; 11 Health and Services Research Unit, Akershus University Hospital, Box 1000, 1478 Lørenskog, Norway

**Keywords:** Hospital, Implementation, Law, Policy, Prevention, Child responsible personnel, Children of ill parents, Children as next of kin, Parental illness

## Abstract

**Background:**

Norway is one of the first countries to require all health professionals to play a part in prevention for children of parents with all kinds of illnesses (mental illness, drug addiction, or severe physical illness or injury) in order to mitigate their increased risk of psychosocial problems. Hospitals are required to have child responsible personnel (CRP) to promote and coordinate support given by health professionals to patients who are parents and to their children.

**Methods:**

This study examined the extent to which the new law had been implemented as intended in Norwegian hospitals, using Fixsen’s Active Implementation Framework. A stratified random sample of managers and child responsible personnel (*n* = 167) from five Hospitals filled in an adapted version of the Implementation Components Questionnaire (ICQ) about the implementation of policy changes. Additional information was collected from 21 hospital coordinators (H-CRP) from 16 other hospitals.

**Results:**

Significant differences were found between the five hospitals, with lowest score from the smallest hopitals. Additional analysis, comparing the 21 hospitals, as reported by the H-CRP, suggests a clear pattern of smaller hospitals having less innovative resources to implement the policy changes. Leadership, resources and system intervention (strategies to work with other systems) were key predictors of a more successful implementation process.

**Conclusions:**

Legal changes are helpful, but quality improvements are needed to secure equal chances of protection and support for children of ill parents.

**Trial registration:**

The study is approved by the Regional Committee on Medical and Health Research Etics South-East (reg.no. 2012/1176) and by the Privacy Ombudsmann.

## Background

There is wide international variation in legislation, policy and practice regarding children with mentally ill parents, ranging from complete lack of provision, stigma and loss of parental rights in some countries, to regional or nationwide preventive child and family policy and legislation in others [1–6]. A considerable body of research has focused on efforts to reduce the transgenerational risk of psychosocial problems among children of ill parents. This includes developing programmes to support children and families where parents are suffering from mental illness [7–10], substance abuse problems [11, 12] or physically illness [13, 14]. Early intervention and prevention have been clearly shown to reduce risks for children. A meta-analysis of 13 individual, group and family interventions found a 40% reduction in the risk of children developing the same mental illness as their parents, by increasing parenting skills and increasing knowledge and strengthening resilience factors among adolescents [15].

In Norway, a new law passed in 2010 requires all health professionals to “help safeguard the need for information and necessary support that minor children (0–18 years) of patients with mental illnesses, drug addiction or severe physical illness or injury may have due to parent’s condition” (Children’s Best Interests’ translation) [16, 17]. Health institutions must comply with the law by having child responsible personnel (CRP) promote and coordinate support given by health professionals to patients in their parental role, and their children.

The new regulations require all health professionals to; a) register dependent children in the patient’s health record, b) have conversations with the parent about children’s need for information and support, c) offer help in family information sharing and conversations with children, d) ensure that children can visit parents at the hospital, e) assess children’s and the family’s needs, and (f) gain parents consent to cooperate with other services in establishing necessary support [16]. This is in line with United Nations Convention on the Rights of the Child [18] stating that children have a right to both participation and protection. Health institutions are required to make plans for education and supervision and develop clinical guidelines and procedures to ensure compliance with the new regulations [17], as well as to establish the required CRPs to support and systematize the work [16].

Numerous barriers to implementing family focused practices have been identified by previous research. These include differences across countries, organizational factors such as lack of resources and inadequate procedures, professional background, cultural and educational factors, such as health professionals’ attitudes, lack of expertise and lack of cooperation, and the availability of families [19–24]. More generally, implementation of best practice guidelines is found to face barriers at individual practitioner level, social context, and organizational and environmental context [25] and it is recommended to tailor implementation strategies to different groups of stakeholders [26].

Leaders play a critical role in creating organizational readiness for change [27], and in developing strategies to support implementation of innovations. Transformational leadership, with leaders who can inspire and motivate the employees, is found to predict implementation of innovative practice [28] and is associated with an innovation climate and more positive staff attitudes to adopt evidence-based practice [29].

This study sought to examine the impact of the mandatory changes in law upon Norwegian health services, and formed part of a large multicentre study, the Children of Ill Parents (CHIP)-study [30] of patients, their partners, and children’s satisfaction with the implementation of the changes in law. Norway is one of the first countries (together with Finland, Sweden and the UK) to require all health professionals to play a part in prevention for children of parents with all kinds of illnesses. This study offers unique insight into the process of a nationwide introduction of new, family focused legislation.

### The framework used in this study

**Fig. 1:**
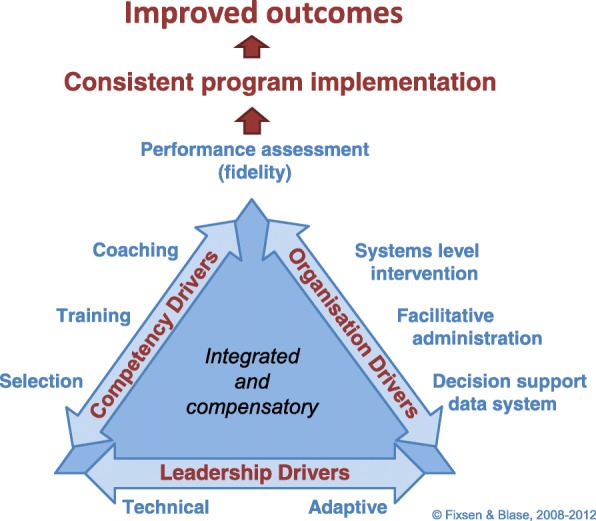
Implementation drivers of practice change, taken with permission, Fixsen and Blase [35]

The Active Implementation Framework (AIF) employed here is based on Fixsen’s review and synthesis of the implementation literature [31], which has been further refined by the National Implementation Research Network (NIRN) [32]. The AIF is measured by the Implementation Components Questionnaire (ICQ) [33]. Implementation is characterized by active and planned efforts to mainstream an innovation within an organization, while dissemination is active and planned efforts to persuade target groups to adopt an innovation [34]. Figure 1 below summarises the drivers shown to be important in the implementation process [35]. These include competency related drivers such as; selection of personnel, training, coaching and performance assessment. Organizational drivers such as facilitative administration must be established to support the new practice development, and decision support data systems need to be changed or improved to be able to collect data on quality improvement. Systems level interventions are strategies to work with other systems or organizations to get support or cooperation, to secure financial and human resources, and to get public support. Finally, a critical driver is leadership. Both technical and adaptive leadership strategies are needed to succeed with implementation and achieve sustainable outcomes [36, 37].

To achieve high quality implementation, researchers recommend establishing specialised implementation teams [37, 38] to oversee the implementation process, establish feedback loops, assess whether the intervention is being used as planned, and promote sustainability.

### The Norwegian strategy for dissemination and implementation

Norway has a public health system, with four regional health authorities (RHF), responsible for ensuring specialist health services are provided to the population in their area. These services are provided through health trusts (HF) and comprise hospitals with inpatient and outpatient services. Private hospitals have agreements with the RHF.

The Ministry of Health and Care Services submits annual requirements to the regional health authorities based on government policy. To support new practice regarding children, a National Competency Network, named “Children’s Best Interests”, was established in 2007 to collect, systematize, and communicate knowledge about children as next of kin/children of ill parents [39].

The Norwegian efforts to secure dissemination of the legal changes in specialist health services comprised; a) a circular [17] b) a commissioning document [40] c) a small budget in 2009 allocated to projects in the regional health authorities [41] d) conferences in all four health regions in 2009–2010, e) training for CRPs and leaders at seven hospitals piloted by Children’s Best Interests, as well as web-based learning resources [39] and learning networks.

Though a National Competency Network was established, implementation was not included in its remit, and their role was more to systematize and disseminate knowledge, instead of being a national implementation team. Initially, there was a lack of implementation support from both the Ministry of Health and Care Services [42] and regional health authorities, with no stable funding of coordinators and infrastructure needed in the hospitals [41, 43]. Based on recommendations from the implementation research [37, 38], we hypothesized that there would be differences in how the law was being implemented.

### Aims

The overarching aim of this study was to examine to what extent the law was being implemented as intended. The first objective was to analyse and compare differences in implementation of changes in law between Norwegian hospitals, based on assessments by managers/leaders and ‘child responsible personnel’. The second was to identify predictors of successful implementation.

## Method

### Design and context

The five hospitals that were the focus of this exploratory and cross-sectional multicentre study serve 34% of the total Norwegian population of 5.2 million people. To get maximum diversity we included five hospitals of different sizes, from three regions across Norway, including both rural and urban areas. The smallest hospital served 136,000 (H1) and the largest (H5) served 493,000 inhabitants. The three remaining hospital served H2: 290,000, H3: 358,000, and H4: 480,000. Hospital 1 is a district hospital providing health services to a large rural area and Hospital 3 and 5 are university hospitals. 

### Participants

The 167 of the 188 participants in this study were recruited from a stratified, randomly selected sample of leaders/managers (L) (technical directors, clinical heads of departments, unit managers) (*n* = 52), child responsible personnel (CRP) (*n* = 110) and hospital coordinators (H-CRP) (*n* = 5) from the above five hospitals. The response rates were 100% for the H-CRPs, 72% for CRPs and 68% for managers. Additional information was collected from H-CRPs (*n* = 21) from 16 other hospitals across Norway, of them two were private hospitals.

Child responsible personnel (1–2 at every unit) are ordinary health professionals selected by their unit manager to promote and coordinate activity in the units. Hospital coordinators (usually 1–2 at the hospital) systematize hospitals total work, or coordinate activity in the departments, e.g. establish procedures, training and supervision. Most H-CRPs hold this role part time (20–50%) as part of another position, with only one H-CRP holding a 100% position.

Of 52 participating leaders 36 were women (69%), with a mean age of 49 years (*SD* = 10). The mean time since they completed education was 21 years for leaders (*SD* = 10), with a mean time in current position of 5 years. Most leaders were nurses (62%), psychologists (17%), social workers (12%), physicians (8%), or others (2%).

Among the 110 child responsible persons (CRP) 97 were women (88%), with a mean age of 47 years (*SD* = 10). Their mean time since completing education was 18 years (*SD* = 10), with a mean duration of 7 years in post. Most of the CRPs were nurses (45%), social workers (34%), psychologists (10%) nurse assistants (7.3%), or staff with other types of higher education (4.5%), e.g. masters, family therapist. The five hospital coordinators (H-CRP) were women, with a mean age of 51 years (*SD* = 4), 24 years (*SD* = 10) since completing education, and on average 3 years in the post (*SD* = 3). Two coordinators were nurses, one a social worker, and two had other types of training. From the 16 additional hospitals, the 21 hospital coordinators (H-CRP) were women, with a mean age of 51 years (*SD* = 9), 24 years (*SD* = 10) post education, and in the post for 6 years (*SD* = 4). Most were nurses (52%), social workers (24%), and other types training (24%). Many of the H-CRPs were highly experienced, and some had acted as champions for children of ill parents for 10–15 years. In sum, the three participants groups mainly consisted of persons that are of the same gender, age, profession and experience, but had different roles in the hospitals.

### Data collection

The data were collected by the first author from June 2013 to March 2014, with the participants filling in a web-based version of the ICQ [33] during a telephone interview. The interviewer was available for additional questions during the completion of the questionnaire.

### Measure

The Implementation Components Questionnaire (ICQ) was first adapted in Norway [33] from an earlier version of the Measures of Implementation Components of the National Implementation Research Network Frameworks by Fixsen et al. [44] and has been shown to have good psychometric validity [33]. The 89-item questionnaire was slightly modified, or reworded, e.g. *PMTO/MST therapist* was replaced by *child responsible personnel*, and *program* was replaced by *changes in law*. Seven questions were added, especially to capture work to collaborate with other systems.

The present study has nine subscales or ‘implementation drivers’, see Fig. 1 and Appendix. The items had five choices of response: *no* = *0*, *sometimes = 1*, and *yes = 2*, in addition to *not relevant* and *I don’t know*, treated as missing. The Cronbach’s alphas of the subscales ranged between 0.74 and 0.93 (Selection 0.74, Training 0.80, Supervision 0.88, Performance 0.86, Data systems 0.80, Administrative support 0.78, Systems Intervention 0.88, Resources 0.81 and Leadership 0.93).

The respondents were also asked four questions about their satisfaction with the implementation process, which were used to create an implementation satisfaction scale; *1) All in all, it is my experience that the work for children as next of kin has been difficult in my unit; 2) The work for children as next of kin is well integrated into my unit; 3) Overall, I experience success in promoting/advocating the interests of children as next of kin; 4) I am satisfied with how the implementation of the legislative amendments has been implemented into my unit)*. These were rated on a 5-point Likert scale, *strongly disagree* was scored as 1, *disagree* = 2, *undecided* = 3, *agree* = 4, *strongly agree* = 5. The Cronbach’s alpha of the implementation satisfaction scale was 0.88.

The participants were asked a question of whether their unit had made changes to better support children visiting their parents, like a better play area or family room.

During the recruitment process for the larger part of the CHIP-study, 594 registration forms were collected, with anonymous data of the number of patients children available for recruitment, controlling whether children were documented in patients’ health records, as required. These data were used as an outcome measure in the present study, to examine if there was any association between the implementation scores and health professionals’ compliance to register dependent children in parent’s health record according to the new regulations.

### Analysis

The statistical analyses were performed with SPSS (version 21). The 96-item adapted version of the ICQ measure was first analysed by scale reliability analysis, suggesting seven items to be deleted before the component analysis. The measure was tested for internal consistency and in exploratory component analyses using Categorical Principal Component analysis (CATPCA) [45]. The new 89-item measure used in this study, had satisfactory psychometric qualities compared to the earlier 89-item version, used by Ogden [33]. However, the 16 items of the systems level interventions scale were best described by a two-dimensional solution, a) System Intervention, b) Resources. The first dimension focuses on the type of collaboration with the outer context *(*e.g. *work to influence external systems so that they have more understanding of the change of legislation; regional authorities and partners like regional health authorities, regional centres of expertise, county councils, universities and politicians in the region)*. The second dimension focuses on whether the resources are sufficient *(*e.g. *the organisation has sufficient time and capacity to lead the work)*.

Descriptive statistics were used to examine the scores of the implementation drivers and the implementation satisfaction scale (Table 1). Mean scores for the five hospitals and the additional group of H-CRPs, in addition to differences between types of personnel were calculated, and differences were examined using ANOVA (Tables 2, and 3) and post hoc *p*-values were corrected with Bonferroni. In analysis including the additional hospitals, differences (ANOVA) between smaller, medium and larger hospitals across Norway, reported by the hospital coordinators, were also examined. Finally, correlation between implementation drivers and satisfaction with the implementation process were calculated (Table 4) before multiple regression analyses were performed to examine predictors of successful implementation (Table 5).

**Table 1:** Descriptive Statistics for Implementation Drivers and Implementation Satisfaction (*N* = 188)

	Items	Number	*M*	*SD*	Skewness		Kurtosis	
					Statistic	*SE*	Statistic	*SE*
Selection	9	188	0.60	.37	.95	.18	−.91	.35
Training	9	187	0.95	.52	.01	.18	−.56	.35
Supervision	13	183	0.96	.55	−.30	.18	−.91	.36
Performance	11	184	0.55	.54	1.17	.18	.67	.36
Data system	8	184	0.98	.58	.15	.18	−.91	.36
Administration	8	185	1.14	.59	−.23	.18	−.92	.36
SystemsInterv.	9	185	0.38	.48	1.56	.18	1.90	.36
Resources	8	184	1.06	.56	−.05	.18	−.90	.36
Leadership	14	188	1.07	.55	−.19	.18	−1.02	.35
Total Implementation	89	188	0.86	.33	−.02	.18	−.76	.35
Implementation Satisfaction	4	188	3.54	.97	−.28	.18	−.68	.35

Implementation Drivers, range 0–2. Implementation Satisfaction, range 1–5

**Table 2:** ANOVA, Mean differences between Hospitals on Implementation Drivers and Implementation Satisfaction (*N* = 188)

	H1 (*n* = 35)		H2 (*n* = 31)		H3 (*n* = 33)		H4 (*n* = 36)		H5 (*n* = 31)		A (*n* = 21)		*p*
	*M*	*SD*	*M*	*SD*	*M*	*SD*	*M*	*SD*	*M*	*SD*	*M*	*SD*	
Selection	0.52	.39	0.60	.44	0.58	.30	0.66	.37	0.63	.41	0.63	0.27	.672
Training	0.87	.59	1.07	.57	1.12	.46	0.95	.48	0.78	.58	0.85	0.33	.105
Supervision	0.54	.51	1.15	.50	1.14	.49	0.90	.55	1.17	.49	0.91	0.44	.000*
Performance	0.39	.56	0.52	.60	0.69	.50	0.60	.54	0.69	.57	0.38	0.31	.084
Datasystem	0.71	.62	1.20	.56	1.16	.48	0.89	.63	1.20	.54	0.67	0.32	.000*
Administration	0.97	.63	1.23	.71	1.24	.47	1.08	.65	1.24	.55	1.09	0.34	.317
SystemsInterv.	0.32	.42	0.20	.44	0.36	.47	0.34	.35	0.30	.45	0.94	0.52	.000*
Resources	0.91	.62	1.16	.49	1.14	.57	1.12	.49	1.12	.60	0.81	0.51	.091
Leadership	0.78	.52	1.14	.48	1.24	.52	1.09	.49	1.23	.60	0.91	0.56	.002*
Total Implementation	0.67	.34	0.91	.29	0.96	.29	0.85	.32	0.93	.37	0.80	0.29	.004*
Implementation Satisfaction	3.25	.97	3.63	.99	3.58	.91	3.53	.93	3.69	1.13	3.64	.82	.492

*A* Additional Hospitals (1–2 Hospital coordinators from 16 other hospitals). Implementation Drivers, range 0–2, Implementation Satisfaction, range 1–5, * *p* < .05

**Table 3:** ANOVA, Mean differences between Types of Personnel on Implementation Drivers and Implementation Satisfaction (*N* = 167)

	L (*n* = 52)		CRP (*n* = 110		H-CRP(*n* = 5)		*df*	*F*	*p*	Post Hoc
	*M*	*SD*	*M*	*SD*	*M*	*SD*				
Selection	1.21	.44	.85	.56	.80	.41	2.163	8.649	.000^*^	L > CRP
Training	.72	.44	.54	.34	.42	.34	2.164	4.552	.012^*^	L > CRP
Supervision	1.17	.49	.87	.57	1.11	.37	2.159	5.116	.007^*^	L > CRP
Performance	.72	.60	.52	.54	.32	.20	2.160	2.632	.075	
Datasystem	1.22	.59	.94	.58	.86	.68	2.160	4.146	.018^*^	L > CRP
Administration	1.15	.59	1.15	.63	1.10	.42	2.161	.018	.982	
SystemsInterv.	.25	.38	.28	.36	1.44	.60	2.161	23.867	.000^*^	H-CRP > L H-CRP > CRP
Resources	1.19	.50	1.05	.59	.93	.54	2.160	1.283	.280	
Leadership	1.29	.44	.99	.56	1.25	.70	2.164	5.651	.004^*^	L > CRP
Total Implementation	.99	.28	.80	.34	.91	.38	2.164	6.275	.002^*^	L > CRP
Implementation Satisfaction	3.73	.87	3.44	1.03	3.55	1.08	2.163	1.529	.220	

*L* Leaders, *CRP* Child Responsible Personnel, *H-CRP* Hospital coordinators. Implementation Drivers, range 0–2, Implementation Satisfaction, range 1–5, * *p* < .05

**Table 4:** Pearson’s bivariate Correlations between the Implementation Drivers and Implementation Satisfaction (*N* = 188)

	1.	2.	3.	4.	5.	6.	7.	8.	9.
1. Selection	1								
2. Training	.43^**^	1							
3. Supervision	.24^**^	.37^**^	1						
4. Performance	.35^**^	.37^**^	.46^**^	1					
5. Data system	.28^**^	.43^**^	.47^**^	.47^**^	1				
6. Administration	.26^**^	.35^**^	.33^**^	.36^**^	.52^**^	1			
7. SystemsInterv.	.06	.03	.08	−.04	−.11	.04	1		
8. Resources	.28^**^	.40^**^	. 37^**^	.26^**^	.40^**^	.41^**^	−.02	1	
9. Leadership	.29^**^	.48^**^	.45^**^	.35^**^	.51^**^	.51^**^	.12	.61^**^	1
DV.Implementation satisfaction	.28^**^	.36^**^	.38^**^	.23^**^	.35^**^	.37^**^	.22^**^	.54^**^	.62^**^

*DV* dependent variable

^**^Correlation is significant at the 0.01 level

**Table 5:** Regression analysis of Implementation Drivers predicting Implementation Satisfaction (*N* = 188)

	*B*	*SE B*	*β*	*t*	*p*
1(Constant)	2.01	.16		12.53	.000
Selection	.28	.18	.11	1.53	.129
Training	−.11	.14	−.06	−.76	.447
Supervision	.18	.13	.10	1.34	.182
Performance	−.17	.13	−.09	−1.28	.202
Data systems	.03	.13	.02	.22	.830
Administration	.13	.13	.08	1.02	.311
SystemsInterv.	.27	.12	.14	2.32	.022*
Resources	.42	.13	.24	3.14	.002**
Leadership	.67	.15	.38	4.34	.000***

R squared = 0.461, * *p* < .05, ** *p* < .01, *** *p* < .001

The hospitals also were compared on law requirements. The number of child responsible personnel per hospital was calculated. ANOVA was used to calculate differences between hospitals establishing play areas and family rooms. The registration forms, with the number of patient’s children found at the recruitment days, compared to documentation of patient’s children (in the patient’s electronic health record) were explored, and descriptive statistics were used to calculate differences between hospitals.

### Attrition and missing values

The aim was to recruit over 70% of both leaders and CRPs for this study. Though the total response rate was 73%, it was more difficult to recruit leaders (68%), than H-CRPs (100%) and CRPs (72%). However, technical directors from all five hospitals participated, as did 9 to 12 other managers from each of the hospitals.

The dataset had very few missing values (10 values). These were replaced by mean values after found missing at random (MCAR, *p* = 0.925). A few items had high scores for “*don’t know*”, but low scores on “*not applicable*”. In the CATPCA analysis, these were treated as missing values, and imputed as an extra category, after found missing completely at random (MCAR, *p* = 0. 998).

## Results

### Descriptive statistics

The mean total implementation score for all respondents was at a medium level, see Table 1. The implementation satisfaction scale had total mean scores slightly over medium level. The skewness and kurtosis were in a normal range [46].

### Differences between the hospitals

ANOVA showed significant differences between the five hospitals on the total implementation score *F*(5,182) = 3.65, *p* = .004, and on three subscales; supervision *F*(5,177) = 7.61, *p* < .001, decision data support systems *F*(5,178) = 5.88, *p* < .001, and leadership *F*(5,182) = 3.89, *p* = .002, see Table 2.

On the total implementation score, post hoc analysis showed that H1 scored significantly lower than three hospitals; H2 (*p* = .038), H3 (*p* = .004), H5 (*p* = .023). On the supervision driver H1 scored significantly lower than *all* other hospitals, with significantly lower scores on the decision data support systems than H2, H3 and H5, and significantly lower scores on leadership than H3 and H4.

Additional information was collected from the 21 hospital coordinators (H-CRP) from 16 other hospitals, in order to support findings from the five hospitals. As expected, the hospital coordinators scored higher on systems intervention *F* (5,179) = 8.31, *p* < .001, than the larger group of personnel from the five hospitals, which reflects the hospital coordinator’s special role. Comparing only the H-CRP at the five study hospitals with the H-CRP in the additional group, there were no significant differences on systems intervention or on the total implementation score.

In analyses including the additional hospitals, we compared answers from the H-CRP (*n* = 26) from smaller sized hospitals (<3000 FTEs, *n* = 8), medium sized hospitals (3–5000 FTEs, *n* = 9), larger sized hospitals (>5000 FTEs, *n* = 9). The group of smaller sized hospitals scored significantly lower on the total implementation scale *F*(2,23) = 7.264, *p* = .004, and on the subscales; leadership *F*(2,23) = 6.569, *p* = .006, resources *F*(2,23) = 3.947, *p* = .034 and supervision *F*(2,23), *p* = .004.

### Differences between types of personnel on implementation drivers and implementation satisfaction

ANOVA showed significant differences between types of personnel on the total implementation score (*p* = .002), and on six subscales, see Table 3, with post Hoc analysis showing that leaders/managers (L) scored significantly higher than child responsible personnel (CRP) on the total implementation score, and on the five subscales; selection (*p* = .012), training (*p* < .001), supervision (*p* = .007), data systems (*p* = .018) and leadership (*p* = .001). An important finding was that hospital coordinators (H-CRP) represent a middle score, as there were no significant differences between H-CRP and L, or between H-CRP and CRP on the total score or the subscales, except for the systems intervention, where H-CRP scored significantly higher than both L and CRP (*p* < .001). There was no significant difference on implementation satisfaction.

### The relationship between implementation satisfaction and implementation drivers

Initially, correlations were calculated between the implementation drivers and the satisfaction variables. This was followed by multiple regression (enter) analysis with the implementation satisfaction as dependent variable and the nine implementation subscales as predictor (independent) variables, see Tables 4 and 5.

Implementation satisfaction was significantly positively associated with all implementation subscales, see Table 4, with the strongest association to leadership (*r* = 0.62) and resources (*r* = 0.54). A multiple linear regression analysis indicated an equation *F* (9,168) = 15.114, *p* < .001 with *R*^2^ of .461, see Table 5. Significant predictors of satisfaction with the implementation process were leadership, resources and systems intervention.

### Comparing hospitals on law requirements

As required, all five hospitals had established plans for education, and developed clinical guidelines and procedures to ensure compliance with the new regulations.

The hospitals had appointed child responsible personnel to support and systematise the work. There were from 21 to 45 CRPs per 100,000 inhabitants, with the two largest hospitals having a smaller number of CRPs per 100,000 (H1: 39, H2: 45, H3: 41, H4: 21, H5: 24). Four of the hospitals had established a hospital coordinator (H-CRP), while Hospital 1 had coordinators at a lower level.

The hospitals had made some changes (e.g play area or family room) to better support children visiting parents at the hospital, with sum score (*M* = 1.47, *SD* = .70, range 0–2). There were no significant differences between the hospitals.

All hospitals had made changes in the data systems to register dependent children. In somatic clinics 51% of children of patients were registered (306 of 595 children), in mental health clinics 61% (882 of 1438 children) and in substance abuse clinics 71% (352 of 496 children). Differences were found in how well children were registered in patients health record, ranging from H1: 51%, H2: 52%, H3: 77%, H4: 50%, to H5: 82%, with the highest registration rate at the two university hospitals.

## Discussion

The overarching aim of this study was to examine to what extent changes in law was implemented as intended. Overall, the five hospitals had implemented change at a medium level with a similar level of satisfaction. When the five hospitals were compared, there were significant differences on the total implementation score, and on three subscales, with the smallest hospital scoring lowest. There were significant differences between types of personnel on the total implementation score, and on six subscales, with child responsible personnel scoring significantly lower than leaders, suggesting that leaders underestimate the implementation challenges. Factors associated with implementation satisfaction were leadership, resources and systems intervention.

### Differences between the hospitals

Hospital 1 – the smallest hospital in the most rural district - scored lowest on the total implementation score and on three subscales; supervision, decision support data system and leadership. This finding was supported in additional analyses in which smaller, medium and larger hospitals, as reported by the hospital coordinators across Norway were compared. The outcome suggests a clear pattern of higher barriers when smaller hospitals as compared to medium and larger hospitals are implementing the changes in law. Meta-analyses of innovation adoption have found that organisational size can be an important factor [47]. Larger organisations might have better structural resources, such as role specialisation and existing knowledge and skills for innovative practice [48] that perhaps leads to greater changes of practice.

However, the hospital with best results was a medium sized university hospital. This hospital selected the manager at a research unit to be hospital coordinator, with the top management as a steering group for the implementation process. The second highest scoring hospital was the only hospital that had employed a new full-time hospital coordinator. This could indicate a better understanding of the challenges of implementation, and the importance of leadership support. The type of person employed to induce change potentially has important implications for future practice change strategies.

Regarding the poorest performing hospital, it should be noted that a previous project (from 2009 to 2011) was not sustained, and the recent funding from government (2013) [49] was not used to establish a hospital coordinator.

In summary, the lack of a “practice change coordinator” in the hospital and lack of leadership support seems to be negatively related to establishing supervision, where Hospital 1 scored significantly lower than the other hospitals. Supervision is found to be important to achieve the skills needed to change own practice, as training alone does not give the necessary changes [31], and recruitment and supervision components can be related to therapists satisfaction with the implementation progress [33].

### Differences between types of personnel

Child responsible personnel scored significantly lower than leaders on the total implementation score, and on the five subscales; recruitment, training, supervision, decision data support systems and leadership. This indicates that managers might underestimate competency challenges among child responsible personnel, and might overestimate the hospitals leadership support. An important finding was that hospital coordinators represent a middle score between the two groups (L and CRP), which indicates that the answers from the additional group of hospitals coordinators can be used as a middle representation of the other hospitals in Norway.

### Predictors of implementation satisfaction

The regression analysis showed that factors associated with implementation satisfaction were leadership, resources and systems intervention. These findings confirm the importance of leadership and resources [36, 47, 50] and highlights leadership’s’ role in establishing the organisational drivers [31]. Making use of data decision support systems can help leadership to follow up on important issues which can slow down the implementation [32, 35].

Systems intervention was the third factor significantly associated with implementation satisfaction. The total score was very low, indicating that most health professionals and managers do not work with other systems. This seems to be a more spesialised activity, with highest scores from the hospital coordinators. The findings highlight the importance of the hospital coordinators, and their role to systematise the hospital’s’ total work together with the leadership and the other child responsible personnel.

### Comparing hospitals on law requirements

Four years after the legal change, all hospitals had made plans for training, developed clinical guidelines and established changes in data systems to register dependent children. However, outcome data of how well children were documented in the health records differed between the five hospitals, with the highest scores from the two university hospitals that scored highest on the total implementation scale.

The Norwegian process of establishing the legal changes in healthcare institutions comprised several dissemination efforts (a memorandum, commissioning document and regional conferences), which could be classified as a dissemination strategy, rather than an implementation strategy. The national competency network offered pilot training, web-based learning resources and learning networks. These efforts were not enough to secure equal chances of children receiving support and protection from the healthcare providers. Especially, there is a need to study the situation at smaller hospitals and consider strategies to support leadership and organisational change [51].

Initially, there was no national funding for coordinators at the hospitals. However, regional and national evaluations from 2011/2012 [41, 43], contributed to changes in the national funding stream from 2013, with more resources to the hospitals [49], to secure and systematize the work. There was also more emphasis on research, one of them this CHIP-study. From 2014, implementation was also included in the remit of Children’s Best Interests, in addition to a steering group with representatives from the regional health authorities [52]. These policy changes demonstrate the importance of establishing “Policy-Practice Feedback Loops” [53], with practice experiences being fed back to policy makers, and being used to make necessary improvements. After preliminary findings from the multicentre study were launched in a report [30], the Directorate of Health made follow-up requirements to the National Competency Network, based on recommendations from the study.

### Strengths and limitations

In this cross-sectional study, implementation is measured once, while it ideally should be measured several times to allow an examination of changes over time [31], and which is needed to measure outcomes like sustainability of new interventions [54]. There are also limitations inherent in cross-sectional research regarding drawing conclusions from regression analysis, as factors can be associated with, rather than predictive of implementation satisfaction. The data collection took several months, which might have led to some differences in the staff’s perception of the implementation process. On the other hand, there was a good response rate (73%) that was consistent across the five hospitals.

Another limitation was that the implementation data, including satisfaction ratings, relied on self-reports which have the potential to be biased. However, a strength was the inclusion of independent outcome data of children documented in patient’s health record. This is also in line with recommendations [33, 48] to include other outcomes, like adoption and penetration within an organisation.

Earlier research has concluded that there is a need for common definitions, measures and tools [34] to study implementations outcomes, as well as to develop better instruments with higher psychometric quality [55]. One strength of this study is that is uses a well-known framework [35], and an earlier piloted instrument [33] to measure implementation. ICQ appears to be a useful measure of implementation of changes in law to safeguard information and help for children of ill parents. However, a weakness is that it is quite long. In the future it might be possible to pare down the measure to three or four items on each subscale and develop a more brief and pragmatic measure. Finally, there are two key strength of this study. It reports on leadership and organisational drivers, which are not commonly empirically examined and reported [31, 56, 57].

## Conclusion

The Norwegian strategy to establish the changes in law comprised mostly dissemination efforts, rather than being an implementation strategy. This strategy was not enough to secure equal chances of protection for children with ill parents. There were clear implementation differences between the five hospitals, especially in relation to supervision, data support systems and leadership, with the smallest hospital in the most rural location scoring lowest. Leadership, resources and systems intervention were key predictors of implementation satisfaction, with hospital coordinators having a key role, collaborating with other services to establish support for children. Outcome data of how well children were documented in the health records differed between the five hospitals, with the highest scores from the two university hospitals, with the highest implementation scores. In summary, the findings indicate that in the hospitals that invest in leadership, resources and systems intervention, the stakeholders will be both more satisfied with the implementation process and more successful in complying with the new law. To strengthen the implementation support, we recommend national, regional and local implementation teams to be established, making use of decision support data systems, and rapid cycle feedback loops for the leadership at all levels to follow up the implementation process. There is also a need to establish routines for performance assessment (adherence or fidelity checks) and national quality indicators.

## Appendix

### Description of subscales/ implementation drivers

The present study has nine subscales or ‘implementation drivers’: selection *(*e.g. *job description for child responsible personnel is clear)*, training *(*e.g. *the hospital trust has made a plan for the training of other health professionals)*, supervision *(*e.g. *child responsible personnel receive supervision, individually or in a group)*, performance assessment *(*e.g. *the individual unit is evaluated in relation to whether they follow the legislative amendments)*, facilitative administration *(*e.g. *a clear management and teams has been established in the hospital trust / division / clinic to work systematically with implementation of the amendments)*, decision support data system *(*e.g. *responsibility for the development of computer systems, measurement and reporting of follow up on children of ill parents is clearly placed in the organization)*, systems intervention *(*e.g. *work to influence external systems so that they have more understanding of the change of legislation; regional authorities and partners like regional health authorities, regional centers of expertise, county councils, universities and politicians in the region)*, resources *(*e.g. *the organisation has sufficient time and capacity to lead the work)*, and leadership (e.g. *leaders within the organisation have continually looked for ways to align practices with the overall mission, values, and philosophy of the organisation)*.

## Abbreviations

AIF: Active Implementation Framework
CATPCA: Categorical Principal Component analysis
CHIP-study: Children of Ill Parents multicentre study
CRP: Child responsible personnel
H: Hospital
H-CRP: Hospital coordinators
HF: Hospital trusts
ICQ: Implementation Components Questionnaire
RHF: Regional health authority
